# Age-related transcriptome changes in Sox2+ supporting cells in the mouse cochlea

**DOI:** 10.1186/s13287-019-1437-0

**Published:** 2019-12-02

**Authors:** Cheng Cheng, Yunfeng Wang, Luo Guo, Xiaoling Lu, Weijie Zhu, Waqas Muhammad, Liyan Zhang, Ling Lu, Junyan Gao, Mingliang Tang, Fangyi Chen, Xia Gao, Huawei Li, Renjie Chai

**Affiliations:** 1 Jiangsu Provincial Key Medical Discipline (Laboratory), Department of Otolaryngology Head and Neck Surgery, Affiliated Drum Tower Hospital of Nanjing University Medical School, No. 321 Zhongshan Road, Nanjing, 210008 China; 2 Research Institute of Otolaryngology, No. 321 Zhongshan Road, Nanjing, 210008 China; 3 Shanghai Fenyang Vision & Audition Center, Shanghai, China; 4 ENT Institute and Otorhinolaryngology Department of Affiliated Eye and ENT Hospital, Key Laboratory of Hearing Medicine of NHFPC, Shanghai Engineering Research Centre of Cochlear Implant, State Key Laboratory of Medical Neurobiology, Fudan University, Room 611, Building 9, No. 83, Fenyang Road, Xuhui District, Shanghai, 200031 China; 5 MOE Key Laboratory for Developmental Genes and Human Disease, State Key Laboratory of Bioelectronics, Co-Innovation Center of Neuroregeneration, Institute of Life Sciences, Jiangsu Province High-Tech Key Laboratory for Bio-Medical Research, Southeast University, Nanjing, 210096 China; 6 Department of Biotechnology, Federal Urdu University of Arts, Science and Technology, Gulshan-e-Iqbal Campus, Karachi, Pakistan; 7 Jiangsu Rehabilitation Research Center for Hearing and Speech Impairment, Nanjing, 210004 Jiangsu China; 8 Department of Biomedical Engineering, Southern University of Science and Technology, Shenzhen, China; 9 Co-Innovation Center of Neuroregeneration, Nantong University, Nantong, 226001 China; 10 Institute for Stem Cell and Regeneration, Chinese Academy of Science, Beijing, China; 11 Beijing Key Laboratory of Neural Regeneration and Repair, Capital Medical University, Beijing, 100069 China

**Keywords:** RNA-seq, Proliferation, Differentiation, Sphere formation, Gene expression

## Abstract

**Background:**

Inner ear supporting cells (SCs) in the neonatal mouse cochlea are a potential source for hair cell (HC) regeneration, but several studies have shown that the regeneration ability of SCs decreases dramatically as mice age and that lost HCs cannot be regenerated in adult mice. To better understand how SCs might be better used to regenerate HCs, it is important to understand how the gene expression profile changes in SCs at different ages.

**Methods:**

Here, we used *Sox2*^*GFP/+*^ mice to isolate the Sox2+ SCs at postnatal day (P)3, P7, P14, and P30 via flow cytometry. Next, we used RNA-seq to determine the transcriptome expression profiles of P3, P7, P14, and P30 SCs. To further analyze the relationships between these age-related and differentially expressed genes in Sox2+ SCs, we performed gene ontology (GO) analysis.

**Results:**

Consistent with previous reports, we also found that the proliferation and HC regeneration ability of isolated Sox2+ SCs significantly decreased as mice aged. We identified numerous genes that are enriched and differentially expressed in Sox2+ SCs at four different postnatal ages, including cell cycle genes, signaling pathway genes, and transcription factors that might be involved in regulating the proliferation and HC differentiation ability of SCs. We thus present a set of genes that might regulate the proliferation and HC regeneration ability of SCs, and these might serve as potential new therapeutic targets for HC regeneration.

**Conclusions:**

In our research, we found several genes that might play an important role in regulating the proliferation and HC regeneration ability of SCs. These datasets are expected to serve as a resource to provide potential new therapeutic targets for regulating the ability of SCs to regenerate HCs in postnatal mammals.

## Introduction

Hair cells (HCs) in the inner ear play a critical role in converting mechanical sound waves into neural signals for hearing and play a critical role in maintaining balance [1, 2]. Multiple studies have reported that HCs in non-mammalian vertebrates can be regenerated in both the auditory and vestibular systems after HC loss and thus lead to the complete recovery of hearing and balance function [3, 4]. Conversely, HCs in the mammalian cochlea can be spontaneously regenerated after damage only to a very limited extent and only in the neonatal cochlea and cannot be regenerated at all in adult animals, and thus in adult mammals, HC damage causes permanent hearing loss [1, 4–10]. Finding a way to regenerate HCs in mammals could possibly represent a cure for sensorineural hearing loss, which still has no treatment options other than prosthetic devices.

In the mouse organ of Corti, HCs and supporting cells (SCs) emerge from the same inner ear prosensory cells. These inner ear prosensory cells start to exit the cell cycle from the apical turn to the basal turn of the cochlea. The apical prosensory cells exit the cell cycle at around embryonic day 12.5 (E12.5), and the basal prosensory cells exit the cell cycle at around E14.5. The inner ear prosensory cells start to differentiate into HCs and SCs beginning at the mid-base of the cochlea at around E13.5 and reaching the rest of the base and up to the apex of the cochlea over the next few days [11]. SCs in the mouse inner ear have also been shown to be a reliable source for regenerating HCs after damage in vitro either through mitotic or direct differentiation [10, 12–15]. Recent studies have demonstrated that the SCs isolated from the neonatal mouse cochlea are competent to form new HCs in culture [10, 16–18], but the ability of SCs to form spheres in suspension cultures decreases about 100-fold during the second and third postnatal weeks [19]. In contrast, the adult mammalian cochlea has almost no HC regeneration capacity, and attempts to stimulate the dormant regenerative capacity have met with very limited success [15, 20]. Multiple factors have been reported to be involved in regulating the process by which SCs regenerate HCs, including factors in the Wnt, Notch, Hedgehog, and STAT3 signaling pathways [10, 21–24]. HC regeneration strategies have only worked at all in the neonatal mouse cochlea, and none has been able to overcome the age barrier in the adult cochlea. An obvious limitation to these previous strategies has been a lack of understanding of the age-related changes in gene expression profiles, and possible age-related genes regulating the proliferation and HC regeneration ability of SCs have not been identified.

Sox2 is a universal stem cell marker, and it is also expressed in neural progenitor cells at different stages of central nervous system development [25]. In the neonatal mouse inner ear, Sox2 labels the SCs that have been shown to be a reliable source for regenerating HCs after damage. In this study, we performed RNA-seq profiling of the Sox2+ SCs isolated from *Sox2*^*GFP/+*^ transgenic mice at four different postnatal time points and determined the age-related differential expression of genes that might be involved in regulating the proliferation and HC differentiation ability of Sox2+ SCs. The Sox2+ SCs we sorted included Hensen’s cells, Deiters’ cells, pillar cells, inner phalangeal cells, and the cells in the greater epithelium ridge. To further analyze the role of these age-related differentially expressed genes, we constructed a protein–protein interaction network using STRING (Search Tool for the Retrieval of Interacting Genes/Proteins). These datasets are expected to serve as a resource to provide potential new therapeutic targets for regulating the ability of SCs to regenerate HCs in postnatal mammals.

## Materials and methods

### Mice and genotyping

*Sox2*^*GFP/+*^ mice were obtained from the Jackson Laboratory (stock no. 17592). Transgenic mice were genotyped using genomic DNA from tail tips by adding 180 μl 50 mM NaOH, incubating at 98 °C for 1 h, and then adding 20 μl 1 M Tris-HCl to neutralize the base. The genotyping primers were as follows: GFP forward: 5′-CAC ATG AAG CAG CAC GAC TT-3′; GFP reverse: 5′-TGC TCA GGT AGT GGT TGT CG-3′.

The cochleae were harvested at P3, P7, P14, and P30. All applicable international, national, and/or institutional guidelines for the care and use of animals were followed. All animal procedures were performed according to protocols approved by the Animal Care and Use Committee of Southeast University and were consistent with the National Institutes of Health Guide for the Care and Use of Laboratory Animals. All efforts were made to minimize the number of animals used and to prevent their suffering.

### Immunofluorescence

The dissected cochleae or the cultured cells were fixed in 4% paraformaldehyde for 1 h at room temperature, washed three times for 3 min with 1× PBST (0.1% Triton X-100 in PBS), and incubated for 1 h at room temperature in blocking medium (1% Triton X-100, 1% BSA, 10% heat-inactivated donkey serum, and 0.02% sodium azide in PBS at pH 7.2). The primary antibody was diluted in PBT-1 (10% Triton X-100, 1% BSA, 5% heat-inactivated goat serum, and 0.02% sodium azide in PBS at pH 7.2) and incubated with the samples overnight at 4 °C. The samples were washed three times for 3 min with 1× PBST, and the secondary antibody diluted in PBT-2 (0.1% Triton X-100 and 1% BSA in PBS at pH 7.2) was added for 1 h at room temperature. The samples were washed again three times with 1× PBST and then mounted on slides in a mounting medium (DAKO, S3023). Cells were imaged with an LSM700 confocal microscope. The antibodies used in this study were anti-myosin7a (Proteus Bioscience, #25-6790, 1:1000 dilution), anti-sox2 (Santa Cruz, #sc-17320, 1:500 dilution), Alexa Fluor 647 donkey anti-goat IgG (Invitrogen, A-21447, 1:500 dilution), and Alexa Fluor 555 donkey anti-rabbit IgG (Invitrogen, A-31572, 1:500 dilution).

### Flow cytometry

The cochleae were dissected in cold 1× HBSS (Gibco) and transferred to 50 μl 1× PBS in 1.5-ml Eppendorf tubes. A total of 50 μl 0.25% trypsin-EDTA (Invitrogen; #25200-056) was added to the tubes, and these were incubated for 8–12 min at 37 °C. The digestion was stopped by the addition of 50 μl trypsin inhibitor (Worthington Biochem, #LS003570), and 200-μl (Eppendorf, #22491245) and 1000-μl (Eppendorf, #22491253) blunt pipette tips were used to triturate the tissues into single cell suspensions. The cells were filtered through a 40-μm strainer (BD Biosciences, 21008-949) to eliminate clumps, and the GFP+ cells were sorted on a BD FACS Aria III flow cytometer (BD Biosciences).

### Sphere-forming assay and differentiation assay

For the sphere-forming assay, the flow-sorted Sox2+ SCs were cultured at a density of 2 cells/μl in Costar ultra-low attachment dishes (Costar, 3473) for 5 days in DMEM/F12 (Gibco, 11330-032), 2% B27 (Invitrogen, 17504-044), 1% N2 (Invitrogen, 17502-048), IGF (50 ng/ml, Sigma, I8779), EGF (20 ng/ml; Sigma, E9644), b-FGF (10 ng/ml, Sigma, F0291), heparan sulfate (20 ng/ml, Sigma, H4777), and 0.1% ampicillin (Sigma, A9518-5G). For the differentiation assay, we used both flow-sorted GFP+ SCs and spheres from the sphere-forming assay. In the cell-differentiation assay, the flow-sorted Sox2+ SCs were cultured at a density of 50 cells/μl on laminin-coated four-well dishes for 10 days in DMEM/F12, 1% N2, 2% B27, EGF (20 ng/ml; Sigma, E9644), IGF (50 ng/ml, Sigma, I8779), heparan sulfate (20 ng/ml, Sigma, H4777), b-FGF (10 ng/ml, Sigma, F0291), and 0.1% ampicillin. In the sphere-differentiation assay, the first-generation spheres were seeded on laminin-coated four-well dishes and cultured for 10 days in DMEM/F12 medium with 1% N2, 2% B27, and 0.1% ampicillin.

### RNA extraction for RNA-seq analysis

Approximately 5000 GFP+ SCs were isolated by FACS and split into three fractions for separate replicates. RNA-seq libraries of FACS-purified cells were generated using the SMART-Seq v4 Ultra Low Input RNA Kit for Sequencing and the Illumina mRNA-Seq Sample Prep Kit. FACS-purified cells were suspended in 10× lysis buffer. First-strand and second-strand cDNA synthesis, adaptor ligation, and PCR amplification were performed using the Illumina mRNA-Seq Sample Prep Kit. SPRI beads (Ampure XP, Beckman) were used in each purification step after RNA fragmentation for size selection. All libraries were analyzed for quality and concentration using an Agilent Bioanalyzer. Sequencing was performed using the Illumina HiSeq2500 150-bp Paired-End Platform, and FASTQ files of paired-end read files were generated.

### Quantitative real-time PCR

We used the RNeasy Micro Kit (QIAGEN, 74004) to extract the total RNA from ~ 20,000 FACS-sorted GFP+ SCs, and the RevertAid First Strand cDNA Synthesis Kit (Thermo, K1622) was used to synthesize cDNA. Real-time PCR was carried out by using the FastStart Universal SYBR Green Master (Rox) (Roche, 04913914001) on a Bio-Rad C1000 Touch thermal cycler. The expression levels of the target genes were normalized to *Gapdh* and the q-PCR primers are listed in Additional file 1.

### Data analysis

The trimmomatic software was used to trim the RNA-seq reads in the FASTQ files. Clean reads were mapped to the mouse reference genome (mm9) using TopHat followed by transcript assembly and differential gene expression analysis using Cufflinks [26]. Genes and transcripts were annotated using the RefGene database (NCBI). Genes with a *p* value of less than or equal to 0.05 were considered significant. Gene ontology (GO) analysis with the functional annotation tool DAVID 6.7 was performed to assess the extent of functional enrichment [27], which determines whether biological processes are enriched within a list of genes. Protein functional association analysis was performed using STRING on genes in top enriched GO categories.

### Statistical analysis

All of the data presented in the text are means ± standard deviations, and we used GraphPad Prism 6 for statistical analysis. For all experiments, *n* represents the number of replicates, and at least three individual experiments were conducted. Two-tailed, unpaired Student’s *t* tests were used to determine statistical significance when comparing two groups, and one-way ANOVA followed by a Dunnett’s multiple comparisons test was used when comparing more than two groups. A *p* value < 0.05 was considered to be statistically significant.

## Results

### Neonatal SCs have higher sphere-forming ability compared with older SCs in vitro

**Fig. 1:**
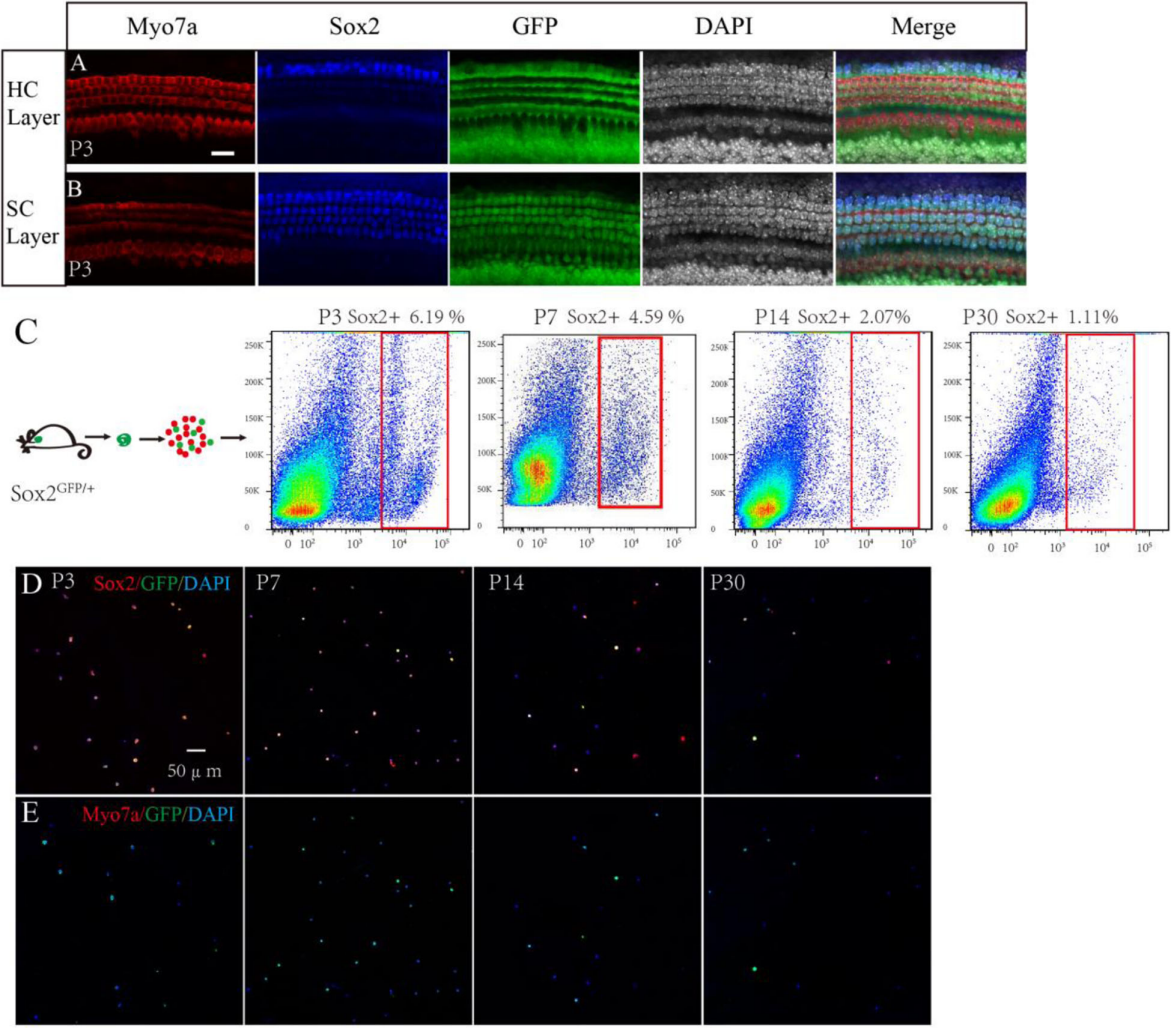
The purity of sorted GFP+ cells. **a** Immunostaining in the HC layer showed no GFP+ cells co-labeled with HCs. **b** In the SC layer, GFP+ cells co-labeled with Sox2+ SCs. **c** Different ages of Sox2^GFP/+^ mouse cochleae were dissected and dissociated into single cells, and the Sox2+ SCs were sorted via flow cytometry. The proportions of Sox2+ cells were 6.19% at P3, 4.59% at P7, 2.07% at P14, and 1.11% at P30. **d**, **e** Immunostaining of flow-sorted Sox2+ SCs of different ages showed a high percentage of Sox2+ and GFP+ cells, and no Myo7a+ cells were found. Scale bars are 20 μm in **a** and **b** and 50 μm in **d** and **e**

First, we performed an immunofluorescence assay to observe the GFP expression pattern in *Sox2*^*GFP/+*^ mice, and we found that GFP was mainly expressed in Hensen’s cells, Deiters’ cells, pillar cells, inner phalangeal cells, and the greater epithelium ridge in the P3 mouse cochlea (Fig. 1a, b). We then used flow cytometry to sort the Sox2+ SCs from cochleae dissected from mice at P3, P7, P14, and P30, and these made up 6.19% of the viable cells in the P3 mice, 4.59% of the viable cells in P7 mice, 2.07% of the viable cells in the P14 mice, and 1.11% of the viable cells in the P30 mice (Fig. 1c). We observed that the proportion of Sox2+ cells gradually decreased with age, and this might be because the increasing ossification with age made the dissection and dissociation of the organ of Corti more difficult at older ages. We then performed immunofluorescence to double confirm the sorted cells and found that at P3 94.9 ± 2.3% and 94.5% ± 2.31% of the sorted cells were Sox2*+* and GFP*+*, respectively, while none of the sorted cells was Myo7a*+* (Fig. 1d, e), suggesting that the flow-sorted cells were almost all Sox2+ SCs and that the sorted cells were highly pure.

**Fig. 2:**
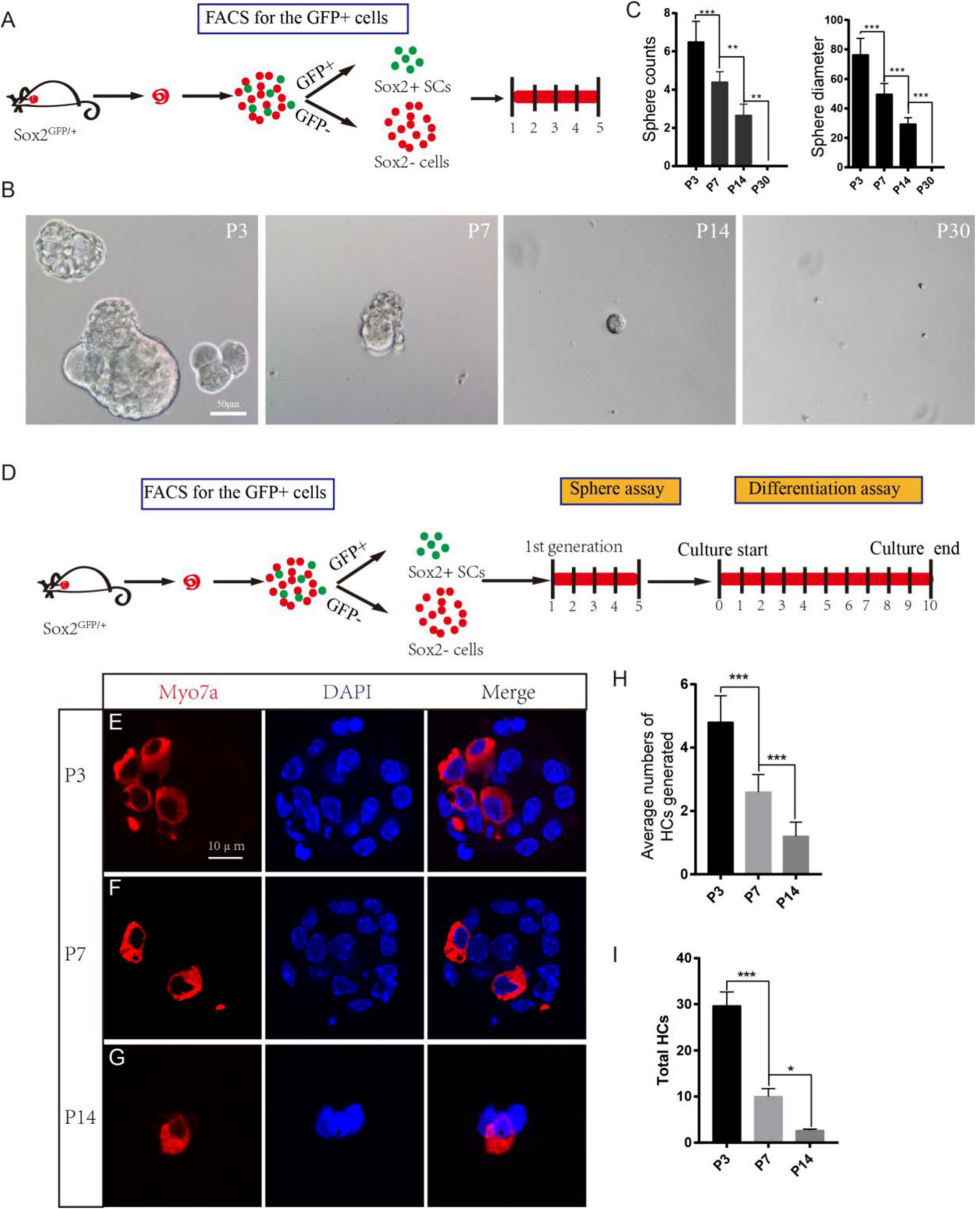
The neonatal SCs have greater sphere-forming ability than the older SCs. **a** The *Sox2*^*GFP/+*^ mice were harvested at P3, P7, P14, and P30. Flow cytometry was used to isolate the different ages of Sox2+ SCs, and these cells were cultured for 5 days. **b**, **c** P3 Sox2+ SCs generated significantly more and larger spheres than P7 and P14 Sox2+ SCs, while the P30 Sox2+ SCs could not form spheres. **d** The cultured cells in the first generation were used for the differentiation assay. **e** The spheres formed by P3 Sox2+ SCs stained with the HC marker Myo7a. **f** The spheres formed by P7 Sox2+ SCs stained with the HC marker Myo7a. **g** The spheres formed by P30 Sox2+ SCs stained with the HC marker Myo7a. **h** The average number of HCs generated by spheres of each age Sox2+ SCs. **i** The total number of HCs generated by P3, P7, P14, and P30 Sox2+ SCs. ***p < 0.001. Scale bars are 50 μm in **b** and 10 μm in **e**–**g**

We next performed a sphere-forming assay using P3, P7, P14, and P30 SCs. A total of 200 isolated cells were plated onto a 96-well ultra-low attachment plate at a density of 2 cells/μl for 5 days (Fig. 2a). We evaluated the proliferation capacity of the SCs by quantifying the numbers and diameters of the spheres. Consistent with previous reports [19], we found that 200 P3 Sox2+ SCs could form around 7 spheres/well and that the diameter of each sphere was more than 70 μm (Fig. 2b). However, the spheres were fewer and smaller from P7 Sox2+ SCs and were even fewer and smaller from P14 Sox2+ SCs (Fig. 2b, c). No spheres were observed from the P30 Sox2+ SCs (Fig. 2b, c). The greater sphere-forming ability of P3 SCs suggests that the neonatal (P3) SCs possess greater proliferation ability than aged (P7, P14, P30) SCs.

In order to further evaluate the HC regeneration ability of these spheres, we isolated the spheres derived from P3, P7, and P14 SCs and differentiated those spheres for 10 days and then immunostained them with the HC marker Myo7a (Fig. 2d). We counted the Myo7a+ HCs in each differentiated sphere and calculated the total Myo7a+ HCs that were generated from the original 200 flow cytometry-isolated Sox2+ SCs. We found that the P3 Sox2+ SC spheres generated significantly more Myo7a+ HCs than the P7 and P14 Sox2+ SC spheres (Fig. 2e–i). In summary, these results support prior findings that neonatal (P3) SCs have a greater capacity to form spheres than aged (P7, P14, P30) SCs and that the spheres formed from neonatal SCs can generate more HCs than spheres formed from aged SCs.

### Neonatal SCs have a greater capacity to regenerate HCs compared with aged SCs in vitro

**Fig. 3:**
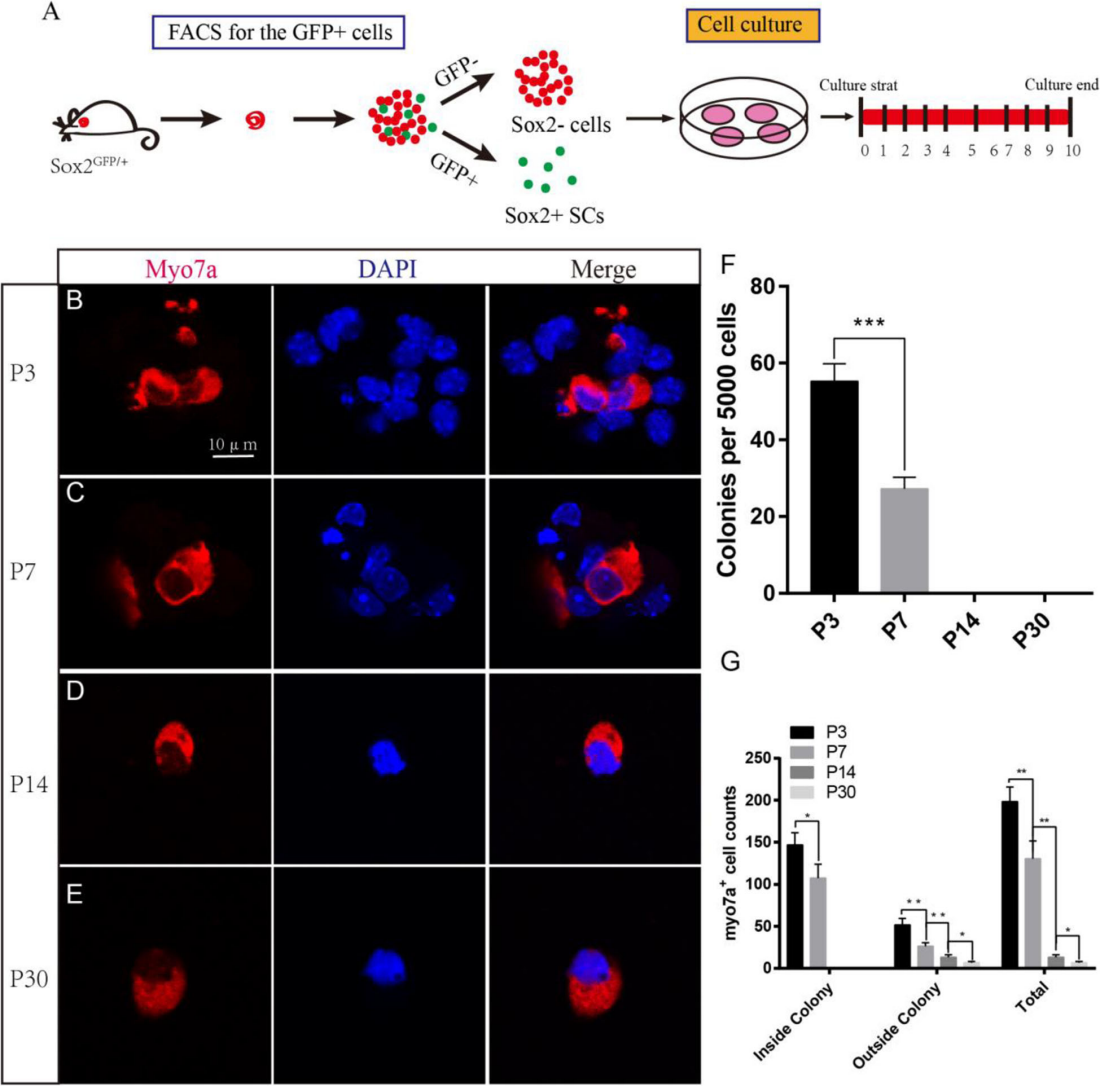
P3 Sox2+ SCs generated more HCs compared with the other three ages of SCs in vitro. **a** We used the FITC channel to sort P3, P7, P14, and P30 Sox2+ SCs, and we cultured the sorted GFP+ cells at 50 cells/μl for 10 days. **b** P3 Sox2+ SCs generated a large number of Myo7a+ cells. **c** P7 Sox2+ SCs also could form colonies and generate Myo7a+ cells. **d**, **e** Both P14 and P30 Sox2+ SCs could not form colonies, but the single cells could generate Myo7a+ cells. **f** P3 Sox2+ SCs formed more Myo7a+ cells compared with P7, P14, and P30 Sox2+ SCs. **g** Both inside and outside of the colony, P3 SCs formed more Myo7a+ cells compared with P7, P14, and P30 Sox2+ SCs. ***p < 0.001. Scale bars are 10 μm in **b**–**h**

Most inner ear cell differentiation occurs during embryonic development, but the neonatal mouse retains a limited ability to regenerate HCs through the differentiation of SCs. This ability is quickly lost, however, and by the first week after birth, there is a notable decline in this regenerative activity. We cultured 5000 isolated Sox2+ P3, P7, P14, and P30 SCs in laminin-coated four-well dishes at a density of 50 cells/μl for 10 days and then immunostained them with the HC marker Myo7a (Fig. 3a). We found that the P3 SCs generated significantly more Myo7a+ colonies than the P7 SCs, while no colonies were seen to develop from P14 and P30 SCs (5000 P3 SCs and P7 SCs generated 146.75 ± 12.71 and 76.5 ± 5.22 HCs inside of the colonies, respectively, *p* < 0.001, *n* = 3) (Fig. 3b–e). At P14 and P30, we only found the HCs outside of the colonies, suggesting that they were directly trans-differentiated from SCs. The total number of Myo7a+ HCs inside and outside of the colonies decreased with age, suggesting that the ability of SCs to regenerate HCs was significantly decreased with age (Fig. 3f).

### RNA-seq analysis of SCs isolated at different ages

**Fig. 4:**
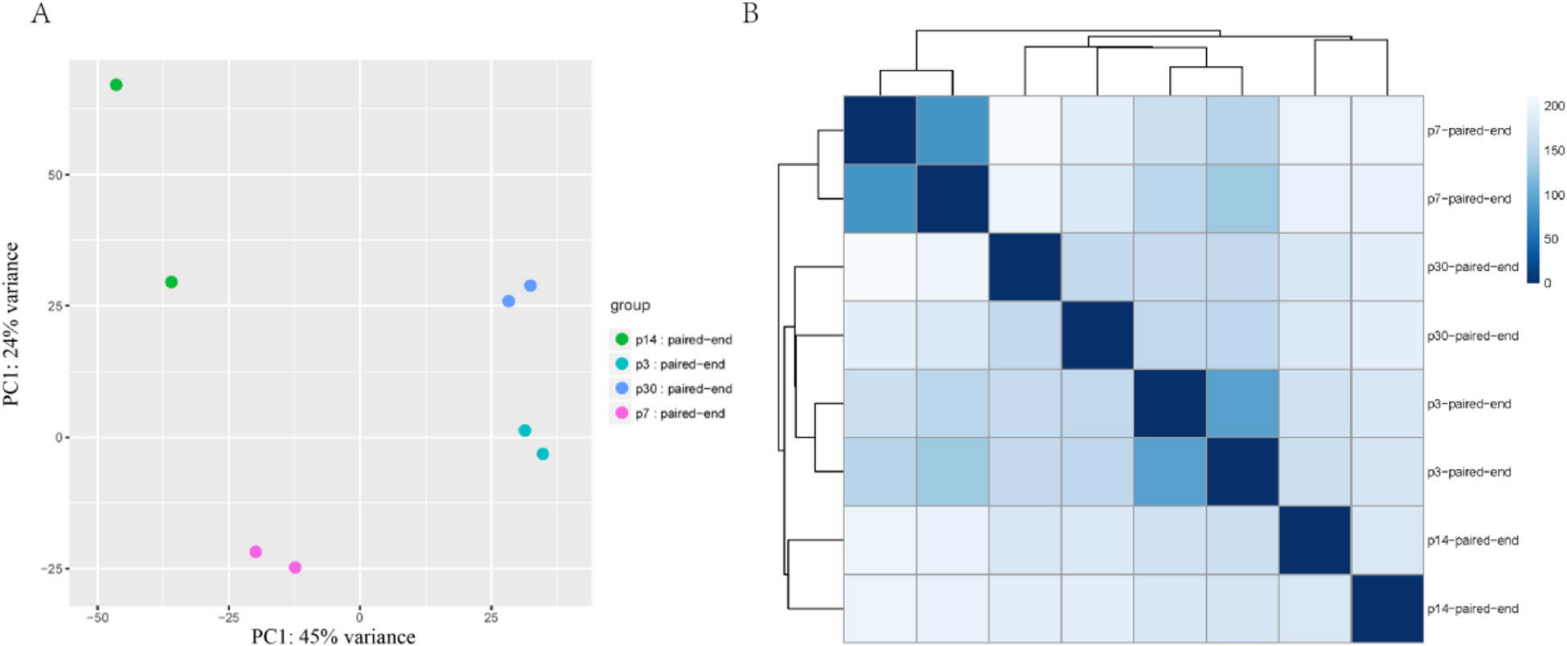
Principal component analysis and sample clustering analysis of expressed genes in P3, P7, P14, and P30 Sox2+ SCs. **a** Principal component analysis of expressed genes for all replicates. Dots in the graph represent replicates. **b** Sample clustering analysis of the replicates

To determine the gene expression profiles of SCs at different ages, RNA-seq analysis was performed on flow cytometry-isolated Sox2+ SCs from P3, P7, P14, and P30 basilar membranes. Three biological replicates were prepared for each time point. After alignment to the reference genome (Mouse mm10, UCSC), the gene expression abundance was normalized to FPKM (fragments per kilobase of transcript per million fragments mapped). We next explored the data set with principal component analysis and sample clustering analysis. Replicates from the same group were well clustered and no outliers were found (Fig. 4). We next carried out pairwise comparison among all time points, and the genes that were differentially expressed within any two groups were marked. In total, we found 1296 differentially expressed genes.

### Cell cycle analysis

**Fig. 5:**
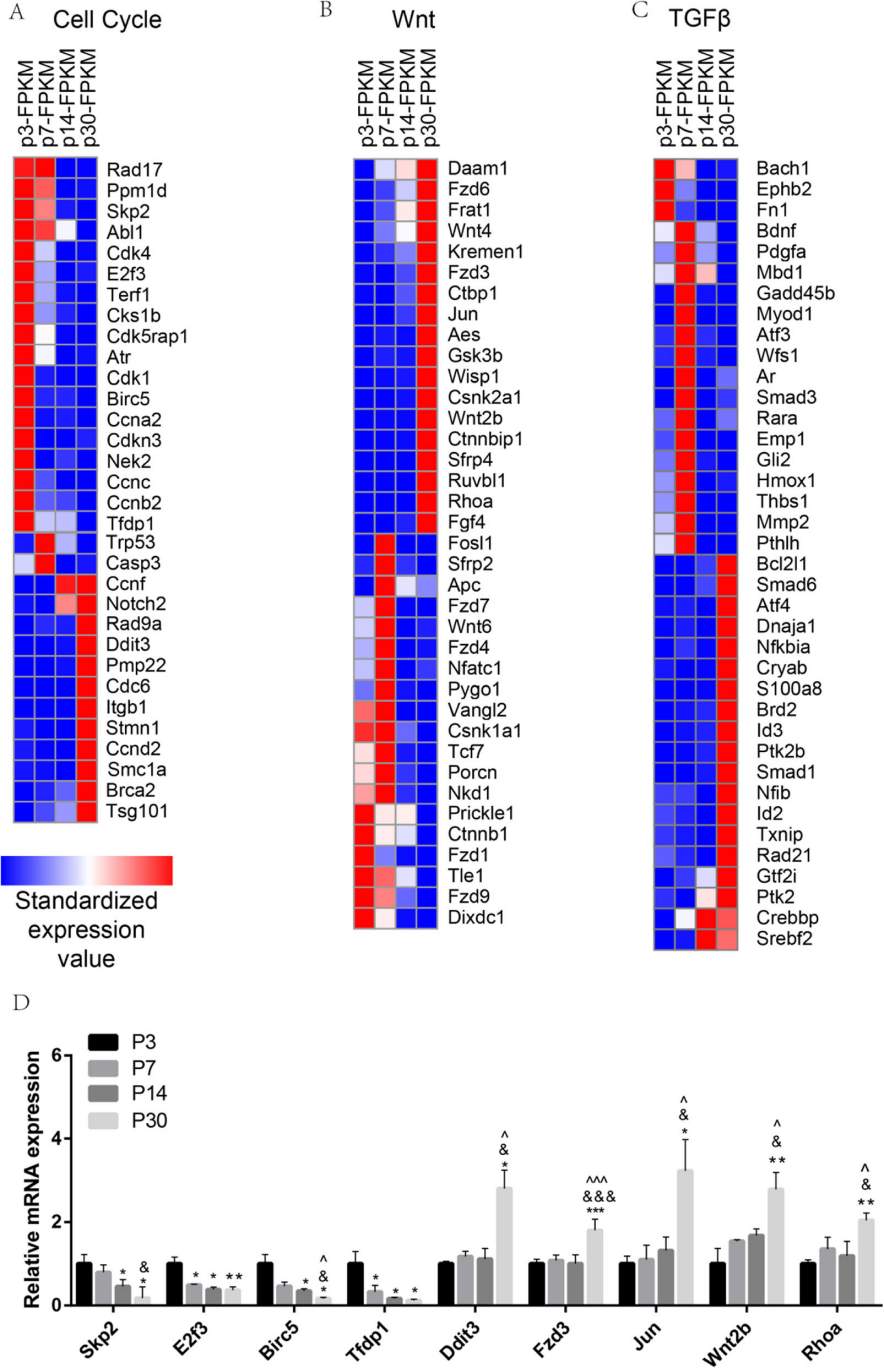
Cell cycle genes, Wnt pathway genes, and TGFβ pathway genes in P3, P7, P14, and P30 Sox2+ SCs. **a** The expression of 72 genes involved in the cell cycle in P3, P7, P14, and P30 Sox2+ SCs. **b** The differentially expressed genes in P3, P7, P14, and P30 Sox2+ SCs that are involved in Wnt signaling pathways. **c** The differentially expressed genes in P3, P7, P14, and P30 Sox2+ SCs that are involved in TGFβ signaling pathways. **d** Quantitative RT-PCR analysis of some cell cycle and Wnt pathway genes that are differentially highly expressed in P3, P7, P14, and P30 Sox2+ SCs as identified by RNA-seq analysis. Student’s paired *t* test; * = P7, P14, and P30 Sox2+ SCs vs. P3 Sox2+ SCs; & = P14 and P30 Sox2+ SCs vs. P7 Sox2+ SCs; ^ = P30 Sox2+ SCs vs. P14 Sox2+ SCs. *p < 0.05, **p < 0.01, ***p < 0.001, ^&^*p* < 0.05, ^&&&^*p* < 0.001, ^*p* < 0.05, ^^^*p* < 0.001

The neonatal Sox2+ SCs had significantly greater proliferation and mitotic HC regeneration ability than the aged SCs; however, the detailed mechanism behind this difference remains unknown. To identify the possible genes regulating the age-dependent cell cycling of SCs, we used RNA-seq analysis to compare the expression of genes regulating the cell cycle and cell proliferation in P3, P7, P14, and P30 SCs. A prior study suggested that over 1000 cell cycle genes might exist in the average mammalian cell [28], some of which had significant expression differences between SCs at different ages. We found that *Rad17*, *Ppm1d*, *Skp2*, *Abl1*, *Cdk4*, *E2f3*, *Terf1*, *Cks1b*, *Cdk5rap1*, *Atr*, *Cdk1*, *Birc5*, *Ccna2*, *Cdkn3*, *Nek2*, *Ccnc*, *Ccnb2*, and *Tfdp1* were highly expressed in the neonatal SCs compared with adult SCs and that *Ccnf*, *Rad9a*, *Ddit3*, *Pmp22*, *Cdc6*, *Itgb1*, *Stmn1*, *Ccnd2*, *Smc1a*, *Brca2*, and *Tsg101* were highly expressed in the adult SCs compared with neonatal SCs (Fig. 5a). Among them, *Skp2* [29–31], *E2f3* [32, 33], *Cdk1* [34, 35], *Birc5* [36], *Ddit3* [37, 38], and *Itgb1* [39] have already been reported in the inner ear. The results of the qPCR were consistent with RNA-seq results, thus confirming the expression difference in the cell cycle genes (Fig. 5d). However, most of the differentially expressed cell cycle genes that we identified at different ages of SCs have not been characterized before in the inner ear and need to be further studied in the future.

### Wnt signaling analysis

The Wnt signaling pathway is a highly conserved pathway and has been reported to be involved in multiple processes including proliferation, cell fate determination, differentiation, and cell protection [40, 41]. In the inner ear, activation of the Wnt signaling pathway is important for HC regeneration and survival [8, 10, 12, 23, 42–47]. To determine which Wnt pathway factors are involved in regulating the age-dependent proliferation and HC regeneration ability of SCs, we measured the expression of over 147 genes, some of which had significant expression differences between SCs at different ages. We found that *Daam1*, *Fzd6*, *Frat1*, *Wnt4*, *Kremen1*, *Fzd3*, *Ctbp1*, *Jun*, *Aes*, *Wisp1*, *Csnk2a1*, *Wnt2b*, *Ctnnbip1*, *Strp4*, *Ruvbl1*, *Rhoa*, and *Fgf4* were significantly upregulated in adult mice compared with neonatal mice, while *Prickle1*, *Ctnnb1*, *Fzd1*, *Tle1*, *Fzd9*, and *Dixdc1* were highly expressed in neonatal mice compared with adult mice (Fig. 5b). Among them, *Jun* [48], *Wnt2b* [49, 50], *Strp4* [51], *Fgf4* [52, 53], *Fzd1* [54], and *Fzd3* [55, 56] have already been reported in the inner ear. We performed qPCR to confirm the RNA-seq data, and the results were consistent with the RNA-seq analysis (Fig. 5d).

### TGFβ signaling analysis

TGFβ signaling plays an important role in inner ear development and HC regeneration [57, 58], but studies of TGFβ signaling in HC regeneration are still limited. To determine which TGFβ pathway factors might be involved in regulating HC regeneration, we examined the expression of TGFβ pathway genes in the mouse genome in P3, P7, P14, and P30 SCs. We found that *Srebf2*, *Crebbp*, *Ptk2*, *Gtf2i*, *Rad21*, *Id2*, *Txnip*, *Nfib*, *Nfkbia*, *Ptk2b*, *Brd2*, *Id3*, *Smad1*, *S100a8*, *Atf4*, *Dnaja1*, *Cryab*, *Bcl2l1*, and *Smad6* were significantly upregulated in adult mice compared with neonatal mice, while *Fn1*, *Ephb2*, and *Bach1* were highly expressed in neonatal mice compared with adult mice (Fig. 5c). Among them, *Ephb2* [59], *Bdnf* [60], and *Pdgfa* [61] have already been reported in the inner ear.

### Notch signaling analysis

**Fig. 6:**
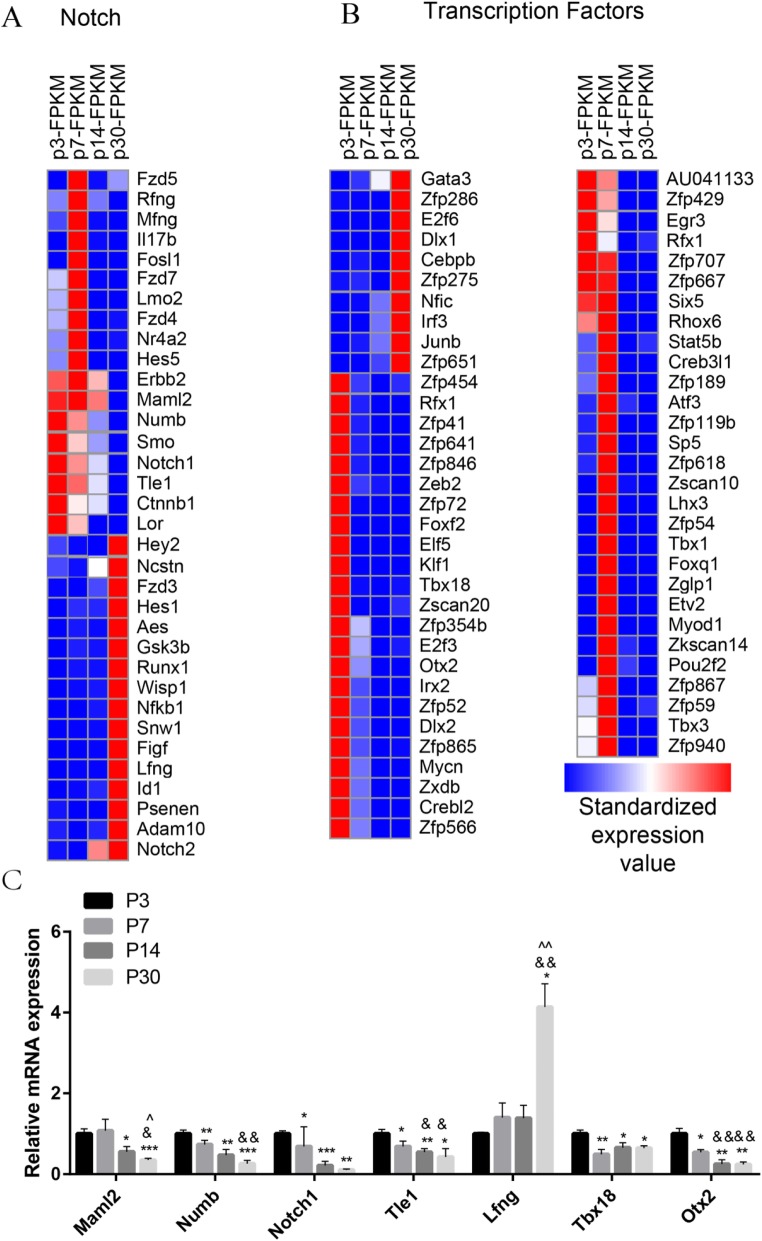
Transcription factor and Notch signaling pathway genes in P3, P7, P14, and P30 Sox2+ SCs. **a** The expression of 96 transcription factor genes in P3, P7, P14, and P30 Sox2+ SCs. **b** The differentially expressed genes in P3, P7, P14, and P30 Sox2+ SCs that are involved in Notch signaling pathways. **c** Quantitative RT-PCR analysis of some transcription factor genes and Notch signaling pathway genes that are differentially highly expressed genes in P3, P7, P14, and P30 Sox2+ SCs as identified by RNA-seq analysis. Student’s paired *t* test; * = P7, P14, and P30 Sox2+ SCs vs. P3 Sox2+ SCs; & = P14 and P30 Sox2+ SCs vs. P7 Sox2+ SCs; ^ = P30 Sox2+ SCs vs. P14 Sox2+ SCs. *p < 0.05, **p < 0.01, ***p < 0.001, ^&^*p* < 0.05, ^&&^*p* < 0.01, ^&&&^*p* < 0.001, ^*p* < 0.05, ^^*p* < 0.01, ^^^*p* < 0.001

Notch signaling plays an important role during the development and patterning of sensory HCs. The activation of Notch signaling promotes the development of progenitor cells but prevents the differentiation of SCs into HCs. Inhibition of Notch signaling or Notch ligands such as *Dll1* and *Jagged2* results in the generation of supernumerary HCs in the mouse inner ear [62–64]. To determine which Notch pathway genes are involved in regulating the age-dependent proliferation and HC regeneration ability of SCs, we measured over 1000 genes, some of which had significant expression differences among SCs at different ages. We found that the expression of *Maml2*, *Numb*, *Smo*, *Notch1*, *Tle1*, and *Lor* decreased with increasing age and that *Hey2*, *Ncstn*, *Hes1*, *Runx1*, *Wisp1*, *Nfkb1*, *Snw1*, *Figf*, *Lfng*, *Id1*, *Psenes*, *Adam10*, and *Notch2* were highly expressed in adult SCs (Fig. 6a). Among these, *Numb* [65], *Smo* [21], *Notch1* [43, 66, 67], *Hey2* [68, 69], *Hes1* [70, 71], *Gsk3b* [72], *Lfng* [73, 74], *Id1* [75, 76], and *Adam10* [77–79] have already been reported in the inner ear. We also performed qPCR to confirm the RNA-seq data, and the results were consistent with the RNA-seq analysis data (Fig. 6c).

### Transcription factor analysis

Transcription factors (TFs) are regulatory proteins that control the expression of targeted genes by binding either to enhancer or promoter regions. TFs are involved in various processes, including inner ear development and HC regeneration. To determine which TFs might be involved in regulating HC regeneration, we examined the expression of 1324 TFs in the mouse genome in P3, P7, P14, and P30 SCs. We found that 9 TF genes (*Zfp286*, *E2f6*, *Dlx1*, *Cebpb*, *Zfp275*, *Nfic*, *Irf3*, *Junb*, and *Zfp651*) were highly expressed in adult mice compared to neonatal mice, while there were 28 TF genes (*Zfp454*, *Zfp41*, *Zfp641*, *Zfp846*, *Zeb2*, *Zfp72*, *Foxf2*, *Elf5*, *Klf1*, *Tbx18*, *Zscan20*, *Zfp354b*, *Otx2*, *Irx2*, *Zfp52*, *Dlx2*, *Zfp865*, *Mycn*, *Zxdb*, *Crebl2*, *Zfp566*, *AU041133*, *Zfp429, Egr3*, *Rfx1*, *Zfp707*, *Zfp667*, and *Six5*) that were highly expressed in neonatal mice compared with adult mice (Fig. 6b). Some of the TF genes that are highly expressed in neonatal SCs have been reported to play roles in promoting HC fate and patterning regulation during inner ear development, including *Rfx1* [80], *Tbx18* [81], *Otx2* [82, 83], *Dlx2* [84], and *Mycn* [85]. We also performed qPCR to confirm the RNA-seq data, and the results were consistent with the RNA-seq analysis data (Fig. 6c). We have identified many TFs that have not been characterized before, and their involvement in the differential regeneration capacity in mouse cochlear SCs at different ages should be investigated in the future.

### Gene ontology analysis of the genes that are differentially expressed in SCs of different ages

**Fig. 7:**
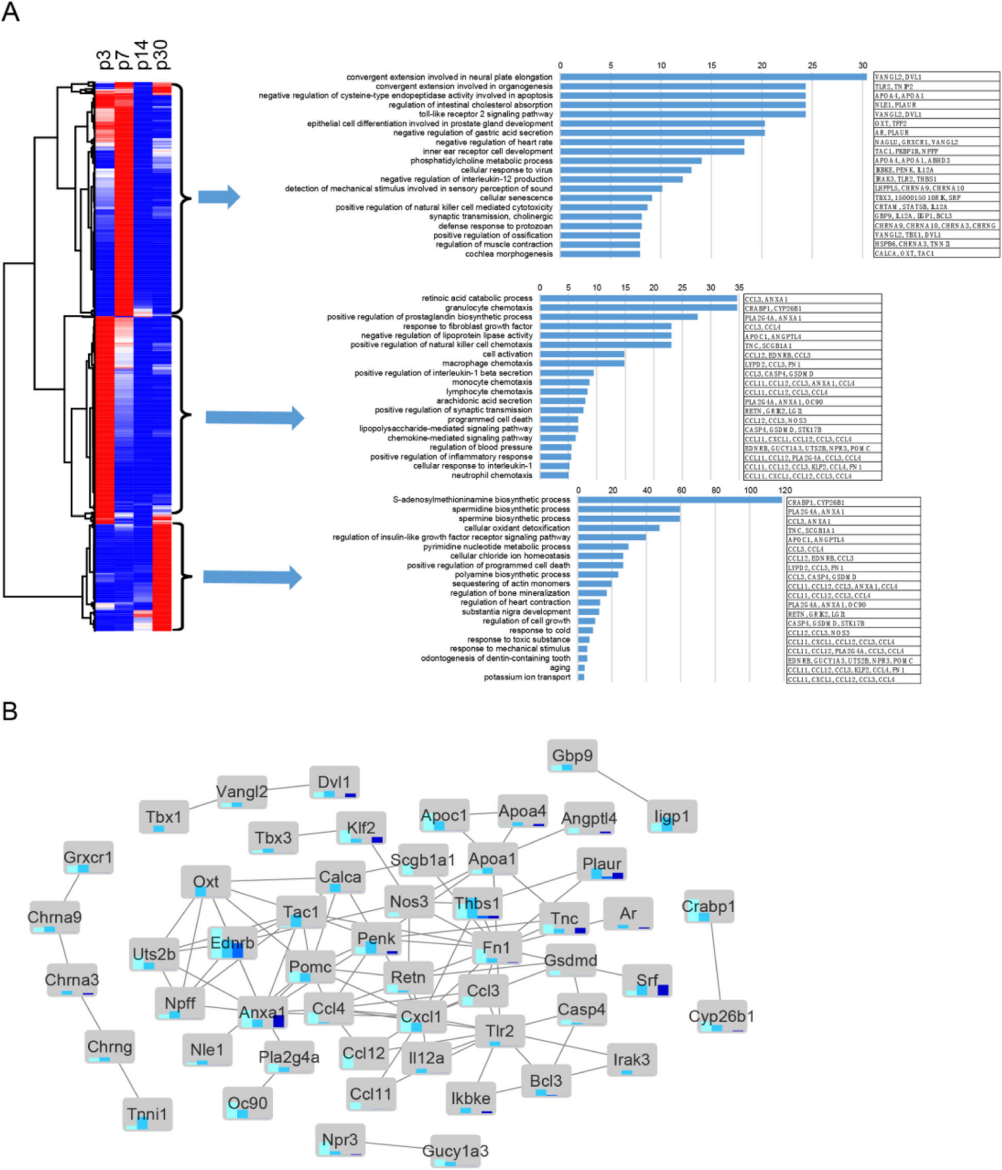
Global comparisons of differentially expressed genes among four time points by hierarchical clustering and gene ontology analysis. **a** Hierarchical clustering of FPKM of all differentially expressed genes. Red denotes above-average expression levels, and blue denotes below-average levels. Each row represents one gene, and each column represents one time point. Gene ontology analysis was performed on the highly expressed gene clusters in the P3, P7, and P30 groups. **b** STRING network analysis of genes present in GO categories

After clustering the expression of all 1296 differentially expressed genes in P3, P7, P14, and P30 Sox2+ SCs in a heatmap (fold change > 2.0, *q* < 0.05), we applied GO analysis to the gene clusters. GO terms with the greatest enrichment fold are shown on the right of Fig. 7a, which also shows the protein interaction network of these GO enriched genes (Fig. 7b). GO analysis was applied to the genes that were upregulated in SCs at different ages (fold change > 2.0, *p* < 0.01). The genes with altered expression in P3 Sox2+ SCs were highly enriched in functional categories such as auditory receptor cell fate determination, neuron fate determination, signaling, and extracellular matrix formation and maintenance. Genes upregulated in P30 SCs were highly enriched in functional categories such as biosyntheic processes and positive regulation of programmed cell death.

## Discussion

Several previous studies have shown that the ability of SCs to regenerate lost or damaged HCs decreases dramatically with age; however, the detailed transcriptome profiles of SCs at different ages have not been studied. Here, we isolated SCs from P3, P7, P14, and P30 mice and compared their transcriptome expression profiles. We identified a set of differentially expressed genes, including cell cycle genes, signaling pathway genes, and TFs, that might be involved in regulating the proliferation and HC differentiation ability of SCs. Most of the differentially expressed genes identified in this study have not been investigated in the inner ear before and need to be further studied in the future.

In order to find the key genes regulating inner ear HC regeneration, our previous studies have reported the transcriptome profiles of SCs or Lgr5+ inner ear progenitors, which are a sub-population of SCs, at different locations and under different treatment conditions [13, 14, 86, 87]. We characterized the transcriptomes of Lgr5+ progenitor cells in the apical and basal turns of the mouse cochlea [14]. Compared with our current results, we found that the cell cycle genes *Ccnc*, *Cdk4*, *Nek2*, and *Skp2* were highly expressed both in the Lgr5+ progenitor cells in the apical turn of the cochlea and in neonatal mouse inner ear SCs. Also, the TF genes *Irx2* and *Zfp667* were highly expressed both in the Lgr5+ progenitor cells in the apical turn of the cochlea and in neonatal mouse inner ear SCs, while *Junb* was highly expressed both in the Lgr5+ progenitor cells in the basal turn of cochlea and in adult mouse inner ear SCs.

We also characterized the transcriptomes of Lgr5+ progenitor cells and other Lgr5− SCs [13]. Compared with our current results, we found that the cell cycle genes *Skp2* and *Terf1* were highly expressed both in the Lgr5+ progenitor cell and in neonatal mouse inner ear SCs, while *Ccnf*, *Notch2*, *Ppm22*, *Ccnd2*, and *Tsg101* were highly expressed both in the Lgr5− SCs and in adult mouse inner ear SCs. The TF gene *Zfp667* was highly expressed both in the Lgr5+ progenitor cells and neonatal mouse inner ear SCs, while *Junb* was highly expressed both in the Lgr5− SCs and adult mouse inner ear SCs. Among Wnt signaling pathway genes, *Wisp1* and *Rhoa* were highly expressed both in the Lgr5− SCs and the adult mouse inner ear SCs.

Next, we characterized the transcriptomes of Lgr5+ progenitor cells with or without neomycin injury to show the damage-induced transcriptome changes in the Lgr5+ progenitors [87]. Compared with our current results, we found that the cell cycle gene *Tfdp1* was highly expressed both in the neomycin-treated Lgr5+ progenitors and neonatal mouse inner ear SCs, while *Stmn1* was highly expressed both in the untreated Lgr5+ progenitors and in adult mouse inner ear SCs. The TF gene *Zfp52* was highly expressed both in the neomycin-treated Lgr5+ progenitors and in neonatal mouse inner ear SCs, while *Junb* was highly expressed both in the untreated Lgr5+ progenitors and in adult mouse inner ear SCs. Among Notch, Wnt, TGFβ signaling pathway genes, *Hes1*, *Ctnnbip1*, *Id2*, and *Id3* were highly expressed both in the untreated Lgr5+ progenitors and in adult mouse inner ear SCs.

Lastly, we characterized the transcriptomes of Lgr5+ progenitor cells and Lgr6+ progenitor cells [86]. Compared with our current results, we found that the TF genes *Ilx2* and *AU041133* were highly expressed both in the Lgr6+ progenitors and in neonatal mouse inner ear SCs; while the cell cycling genes *Rad17* and *Skp2* were highly expressed both in the Lgr5+ progenitors and in neonatal mouse inner ear SCs. Among Notch signaling pathway genes, *Maml2* was highly expressed both in the Lgr6+ progenitors and in neonatal mouse inner ear SCs, while *Hey2*, *Hes1*, and *Id1* were highly expressed both in the Lgr5+ progenitors and in adult mouse inner ear SCs. These candidate genes might play important roles in regulating HC regeneration in the inner ear.

### Cell cycle analysis

Among the differentially expressed cell cycle-related genes, *Skp2*, *E2f3*, *Cdk1*, *Birc5*, *Ddit3*, and *Itgb1* have been reported in the inner ear before*. Skp2* is an F-box protein that regulates the G1 to S transition by controlling the stability of several G1 regulators, including p27, and it is expressed in the auditory epithelia and neurons at early stages of development. In the mature auditory epithelium, overexpression of *Skp2* alone can induce SC proliferation but cannot induce new HC formation, while overexpression of *Skp2* combined with overexpression of *Atoh1* generates new HCs [29–31]. This suggests that regulation of HC regeneration requires multi-gene coordination. *Skp2* is also highly expressed in tumor cells and promotes cell proliferation [88–90]. *E2f3* is a member of the E2F transcription factor family and is involved in regulating cell proliferation. In isolated human islets, it can induce proliferation of β cells [91]. *E2f3* is barely expressed in the inner ear, but its expression increases in outer HC nuclei upon excessive noise exposure [32, 33]. *Cdk1* is ubiquitously expressed throughout the organ of Corti and spiral ganglion cells, and inhibition of *Cdk1* and other cyclin-dependent kinases can induce differentiation of supernumerary HCs and Deiters’ cells in the developing organ of Corti in cultured rat cochleae [34, 35]. *Birc5* is expressed during embryonic development and cannot be detected in most terminally differentiated tissues, and it is also highly expressed in many tumors such as pancreatic ductal adenocarcinoma [92]. *Birc5* is widely expressed in the organ of Corti, and it provides protection against ototoxin-induced cytotoxicity [36]. *Ddit3* is an endoplasmic reticulum stress marker gene. In the animal model of acute hearing loss, the expression of *Ddit3* is upregulated in the lateral wall of the cochlea, and this high expression of *Ddit3* might lead to hearing loss because of endoplasmic reticulum stress [37, 38]. *Itgb1* is involved in the regulation of cell migration and invasion of hepatoma carcinoma, breast cancer, and gallbladder cancer [93–95]. It is expressed throughout the otic area, including the fusion plate epithelium and the periotic mesenchyme [39]. *Rad17*, *Ppm1d*, *Abl1*, *Cdk4*, *Terf1*, *Cks1b*, *Cdk5rap1*, *Atr*, *Ccna2*, *Cdkn3*, *Nek2*, *Ccnc*, *Ccnb2*, *Tfdp1*, *Ccnf*, *Rad9a*, *Pmp22*, *Cdc6*, *Stmn1*, *Ccnd2*, *Smc1a*, *Brca2*, and *Tsg101* have not been reported previously in the inner ear and need to be further studied in the future.

### Wnt signaling analysis

Among the differentially expressed Wnt signaling-related genes, *Jun*, *Wnt2b*, *Strp4*, *Fgf4*, *Fzd1*, *Fzd3*, and *Fzd6* have been previously reported in the inner ear. *Jun* has been implicated in the regulation of cell proliferation, differentiation, and apoptosis. It plays a critical role during inner ear development by mediating apoptosis through the JNK pathway [48]. *Wnt2b* is expressed in the endolymphatic duct; however, the role of *Wnt2b* in inner ear development has not been reported [49, 50]. *Sfrp4* is a Wnt pathway inhibitor that is involved in many diseases including obesity, type 2 diabetes, cancer, and psoriasis [96]. In the inner ear, *Sfrp4* can be directly targeted by miR-124 to regulate HC differentiation and polarization in the organ of Corti [97]. *Fgf4* is present in many cancerous and noncancerous tissues, indicating that *Fgf4* plays an important role in cell differentiation and proliferation [98]. In zebrafish, *Fgf4* can be mediated by miR-194 to regulate the development and differentiation of sensory patches [52, 53]. Frizzled signaling is involved in diverse tissue closure processes, and defects in frizzled signaling result in some of the most common congenital anomalies in humans. In the organ of Corti at E18, *Fzd1* is weakly expressed in the three outer rows of sensory HCs and is strongly expressed in the flanking non-sensory epithelial cells and the underlying phalangeal and pillar cells, and *Fzd1* mutations cause mis-orientation of inner ear sensory HCs [54]. *Fzd3* and *Fzd6* are key regulators of planar cell polarity in the mammalian cochlea. In the inner ear, both *Fzd3* and *Fzd6* are localized on the lateral faces of sensory and SCs in all sensory epithelia, and this localization overlaps with *Vangl2* and suggests that *Fzd3* and *Fzd6* might play an important role in the planar polarity of HCs because *Vangl2* plays an important role in regulating hair bundle orientation [55, 56, 99]. This suggests that different Frizzled genes in the inner ear have different functions. Although *Jun*, *Wnt2b*, *Strp4*, *Fgf4*, *Fzd1*, *Fzd3*, and *Fzd6* have been previously reported in the inner ear, the function of these genes in HC regeneration still need to be studied further. *Daam1*, *Frat1*, *Wnt4*, *Kremen1*, *Ctbp1*, *Wisp1*, *Csnk2a1*, *Ctnnbip1*, *Ruvbl1*, *Rhoa*, *Prickle1*, *Ctnnb1*, *Tle1*, *Fzd9*, and *Dixdc1* have not been reported previously in the inner ear and need to be further studied in the future.

### TGFβ signaling analysis

Among the differentially expressed TGFβ signaling-related genes, *Ephb2*, *Bdnf*, and *Pdgfa* have previously been reported in the inner ear. *Ephb2* is a member of the largest group of transmembrane receptor tyrosine kinases, and deletion of *Ephb2* leads to vestibular dysfunction because of the reduced production of endolymph [59]. *Bdnf* acts as a nerve growth factor, and it promotes the growth and survival of neurons in the central and peripheral nervous systems [100]. In the inner ear, it supports spiral ganglion neuron survival [60]. *Pdgfa* is a growth factor with restricted otic expression, and it overlaps with *Fgf16* in the anterior and posterior cristae in the chick inner ear [61]. *Srebf2*, *Crebbp*, *Ptk2*, *Gtf2i*, *Rad21*, *Id2*, *Txnip*, *Nfib*, *Nfkbia*, *Ptk2b*, *Brd2*, *Id3*, *Smad1*, *S100a8*, *Atf4*, *Dnaja1*, *Cryab*, *Bcl2l1*, *Smad6*, *Fn1*, and *Bach1* have not been reported previously in the inner ear and need to be further studied in the future.

### Notch signaling analysis

Among the differentially expressed Notch signaling-related genes, *Numb*, *Smo*, *Notch1*, *Hey2*, *Hes1*, *Gsk3b*, *Lfng*, *Id1*, and *Adam10* have previously been reported in the inner ear. *Numb* is a cell fate determinant gene that regulates cardiac progenitor cell differentiation and cardiac morphogenesis [101]. In the auditory epithelium, *Numb* expression has different patterns, which suggests that *Numb* plays an important role in cochlear development [65]. *Smo* encodes a membrane protein that is essential for the transduction of Hedgehog signals into the cytoplasm. Activation of *Smo* inhibits prosensory cell differentiation into HCs or SCs and maintains their properties as prosensory cells, and conditional knockout of the *Smo* gene in the cochlea delays HC and SC differentiation in the apical region [21]. *Notch1* is the primary Notch receptor expressed in the mouse inner ear, and activation of *Notch1* in developing auditory HCs causes profound deafness, while deletion of *Notch1* leads to limited mitotic HC generation [43, 66]. *Hey2* is a putative Notch target gene and functions in cell fate specification. *Hey2* is expressed in the cochlear epithelium prior to terminal differentiation, and its overexpression overlaps with that of *Hes1* in the developing cochlea. The genetic inactivation of *Hey2* leads to increased numbers of mis-patterned inner HCs and outer HCs [70, 71], and activation of *Hey2* by FGF signaling blocks HC differentiation [68, 69]. *Gsk*3 plays an important role in the regulation of apoptosis and proliferation in the inner ear, and activation of *Gsk*3 causes the release of inflammatory factors that can eventually lead to hearing loss, while inactivation of *Gsk*3 increases the total number of HCs [72, 102]. The *Lfng* gene is expressed in non-sensory SCs in the mouse cochlea, but there is no noticeable effect on HC differentiation in *Lfng* mutant mice. However, mutation of *Lfng* suppresses the effects of the *Jag2* mutations on inner HCs [73, 74]. *Id1* is able to prevent differentiation of pluripotent cells, and in bone marrow transplantation assays, reducing *Id1* enhanced hematopoietic stem cells’ self-renewal potential [103]. *Id1* is expressed within the cochlear duct in a pattern that is consistent with a role in the regulation of HC development. However, there is no hearing deficit in the absence of the *Id1* gene, and the reason for this might be compensatory effects by other *Id*s like *Id3*, which has a similar expression pattern as *Id1* in the cochlea [75, 76]. *Adam10* is abundantly expressed in the brain and is linked to epilepsy, Alzheimer’s disease, Hunting’s disease, and developmental disorder Fragile X syndrome because of its role in regulating the activity of excitatory synapses [104, 105]. *Adam10* is also expressed in the cochlea and vestibule, and inhibition of *Adam10* after HC loss increases the proliferation of SCs in the vestibular system [77–79]. Although *Numb*, *Smo*, *Notch1*, *Hey2*, *Hes1*, *Gsk3b*, *Lfng*, *Id1*, and *Adam10* have been reported in the inner ear, the functions of some of these genes in HC regeneration still need to be further studied. *Maml2*, *Tle1*, *Lor*, *Ncstn*, *Runx1*, *Wisp1*, *Nfkb1*, *Snw1*, *Figf*, *Psenes*, and *Notch2* have not been reported previously in the inner ear and need to be further studied in the future.

### Transcription factor analysis

Among the differentially expressed TFs, *Rfx1*, *Tbx18*, *Otx2*, *Dlx2*, and *Mycn* have previously been reported in the inner ear. *Rfx1* has an important function in brain tumors and sensorineural hearing loss. Together with *Atho1*, *Rfx1/3* can induce HC-like cell differentiation, and the conditional knockout of *Rfx1/3* leads to severe hearing loss and OHC damage [80, 106, 107]. *Tbx18* is a critical TF for cell proliferation and cell fate determination, and it is also a target gene of the Hippo pathway [108]. The expression of *Tbx18* during inner ear development is restricted to the sub-region of the otic mesenchyme that is fated to differentiate into fibrocytes, and *Tbx18*-deficient mice show profound deafness and a complete disruption of the endocochlear potential that is essential for the transduction of sound by sensory HCs [81]. *Otx2* is a regulator of embryonic development and embryonic neurogenesis. And it plays a role in brain, craniofacial, and sensory organ development [109, 110]. In the inner ear, *Otx2* can induce *Hes5* and lead to the differentiation of both cochlear and macular neuroepithelium [82, 83]. In the chick inner ear, the expression of *Dlx1* and *Dlx2* during the later stages of inner ear morphogenesis is limited to cochlear and vestibular nerves, and expression levels are lower than in the early stages of morphogenesis [84]. *Mycn* is a critical factor in the development of the central and peripheral nervous systems [111]. In humans, the mutation of *Mycn* can cause structural and functional abnormalities of the inner ear [85]. *Zfp286*, *E2f6*, *Dlx1*, *Cebpb*, *Zfp275*, *Nfic*, *Irf3*, *Junb*, *Zfp651*, *Zfp454, Zfp41*, *Zfp641*, *Zfp846*, *Zeb2*, *Zfp72*, *Foxf2*, *Elf5*, *Klf1*, *Zscan20*, *Zfp354b*, *Irx2*, *Zfp52*, *Zfp865*, *Zxdb*, *Crebl2*, *Zfp566*, *AU041133*, *Zfp429*, *Egr3*, *Zfp707*, *Zfp667*, and *Six5* have not been reported previously in the inner ear and need to be further studied in the future.

## Conclusion

Consistent with previous reports, in this study we also found that neonatal SCs have significantly greater proliferation and HC regeneration ability than adult SCs. We systematically investigated the transcriptome differences between P3, P7, P14, and P30 SCs and identified several significantly differentially expressed genes that might regulate the proliferation and HC regeneration capacity of SCs of different ages. The transcriptomes of different ages of SCs reported here establish a framework for future characterization of the genes that regulate the proliferation and HC regeneration ability of SCs, and these genes might represent new therapeutic targets for HC regeneration.

## Supplementary information

**Additional file 1.** q-PCR primers

## Data Availability

The datasets during and/or analyzed during the current study are available from the corresponding author on reasonable request.

## Abbreviations

E: Embryonic day
GO: Gene ontology
HC: Hair cell
P: Postnatal day
SC: Supporting cell
STRING: Search Tool for the Retrieval of Interacting Genes/Proteins
TFs: Transcription factors
