# Supplementary material for: Age-related transcriptome changes in Sox2+ supporting cells in the mouse cochlea
Source: Stem Cell Res Ther. 2019 Dec 2;10:365. doi: 10.1186/s13287-019-1437-0 (PMC6889721; doi:10.1186/s13287-019-1437-0)
Supplement: Supplementary file 1 — Additional file 1. q-PCR primers [file 13287_2019_1437_MOESM1_ESM.docx]

Table1.

|  | Forward Sequence (5' -> 3') | Reverse Sequence (5' -> 3') |
| --- | --- | --- |
| Skp2 | ATGGACTGCTCTCAAACCTCG | CCTGGAAAGTTCTCCCGACTAA |
| Birc5 | GAGGCTGGCTTCATCCACTG | CTTTTTGCTTGTTGTTGGTCTCC |
| Tfdp1 | TTGAAGCCAACGGAGAACTAAAG | TGGACTGTCCGAAGGTTTTTG |
| E2f3 | AAACGCGGTATGATACGTCCC | CCATCAGGAGACTGGCTCAG |
| Jun | CCTTCTACGACGATGCCCTC | GGTTCAAGGTCATGCTCTGTTT |
| Ddit3 | CTGGAAGCCTGGTATGAGGAT | CAGGGTCAAGAGTAGTGAAGGT |
| Fzd3 | ATGGCTGTGAGCTGGATTGTC | GGCACATCCTCAAGGTTATAGGT |
| Wnt2b | CCGACGTGTCCCCATCTTC | GCCCCTATGTACCACCAGGA |
| Rhoa | AGCTTGTGGTAAGACATGCTTG | GTGTCCCATAAAGCCAACTCTAC |
| Numb | AAAGCAGTGAAGGCCGTTCT | GTTTTCTCGTCCACAACTCTGAG |
| Maml2 | TTTCCTTGGCTAACTCTGCAC | CCCTGTTTGCTCCTGATACTG |
| Tle1 | CCAGTACCTCTCACGCCTCA | GCCCACTCAGAGCACTAGAC |
| Notch1 | GATGGCCTCAATGGGTACAAG | TCGTTGTTGTTGATGTCACAGT |
| Tbx18 | GTACCTGGCTTGGCACGAC | GCATTGCTGGAAACATGCG |
| Lfng | CGAGGTGCATAGCCTCTCC | GCGAGGGGACAGAACTTCG |
| Otx2 | GTACCTGGCTTGGCACGAC | GCATTGCTGGAAACATGCG |
| GAPDH | AGGTCGGTGTGAACGGATTTG | TGTAGACCATGTAGTTGAGGTCA |
